# ANXA2 suppresses antiviral immunity by impeding STING Golgi translocation and disrupting the TBK1/IKKε-IRF3 axis

**DOI:** 10.1128/jvi.02081-25

**Published:** 2026-05-11

**Authors:** Hongyang Liu, Mengdi Xue, Chunying Feng, Jimin Yu, Guangqiang Ye, Kunli Zhang, Li Huang, Changjiang Weng

**Affiliations:** 1 Division of Fundamental Immunology, State Key Laboratory of Animal Disease Control and Prevention, Harbin Veterinary Research Institute of Chinese Academy of Agricultural Sciences 111613 , Harbin, China; 2 Heilongjiang Provincial Key Laboratory of Veterinary Immunology, Harbin, Heilongjiang, China; University of Virginia, Charlottesville, Virginia, USA

**Keywords:** Annexin A2, S100A10, transport of STING, HSV-1, cGAS-STING

## Abstract

Upon viral infection, the cGAS-STING axis is pivotal for initiating type I interferon (IFN) production against DNA viruses. In this study, we identified Annexin A2 (ANXA2) as a novel and potent negative regulator of this antiviral pathway. Overexpression of ANXA2 inhibits herpes simplex virus-1 (HSV-1)-induced IFN-β production, thereby promoting viral replication, whereas ANXA2 deficiency enhances IFN responses and restricts HSV-1 replication. Mechanistically, ANXA2 operates through a dual mechanism. First, it interacts with the transmembrane domain of STING and inhibits its agonist-triggered translocation from the endoplasmic reticulum to the Golgi apparatus, a step essential for STING activation. Second, ANXA2 binds to the nuclear export signal of IRF3, competing with the kinases TBK1 and IKKε for IRF3 interaction. This disrupts the formation of the TBK1-IKKε-IRF3 complex, thereby suppressing IRF3 phosphorylation and nuclear translocation. This function is independent of its canonical binding partner S100A10 but requires ANXA2’s calcium-binding capacity. Crucially, *in vivo* studies using *Anxa2*^⁻/⁻^ mice confirmed that ANXA2 deficiency enhances type I IFN production, limits viral replication, and improves survival following HSV-1 challenge. Collectively, our findings unveil a multifaceted role for ANXA2 in dampening host antiviral immunity by targeting both the STING trafficking and the TBK1/IKKε-IRF3 activation steps, highlighting it as a potential target for immunomodulatory antiviral strategies.

**IMPORTANCE:**

The cGAS-STING axis is essential for host resistance to DNA virus infections by regulating type I interferon (IFN) production. This study is significant because it identifies Annexin A2 (ANXA2) as a previously unrecognized and potent negative regulator of the host antiviral response against DNA viruses like herpes simplex virus-1. The findings demonstrate that ANXA2 suppresses type I interferon production through a dual mechanism: by inhibiting the critical transport of STING to the Golgi apparatus and by disrupting the kinase activity of TBK1/IKKε required for IRF3 activation. This role is independent of its known partner S100A10, revealing a novel function for ANXA2. The validation in knockout mice, which showed enhanced IFN production and restricted viral replication, confirms its physiological importance *in vivo*. By pinpointing ANXA2 as a key inhibitor of the cGAS-STING pathway, this research not only advances our understanding of immune regulation but also identifies ANXA2 as a potential therapeutic target for modulating antiviral defenses.

## INTRODUCTION

Innate immunity is the first line of defense against pathogenic microorganism infections and plays key roles in regulating host antiviral responses to eliminate pathogens. Upon pathogen infection, pattern recognition receptors (PRRs) can recognize pathogen-associated molecular patterns, including viral DNA, viral RNA, and surface glycoproteins, to activate host antiviral responses (1). The known PRRs include toll-like receptors (TLRs), retinoic acid-inducible gene I (RIG-I)-like receptors (RLRs), nucleotide-binding domain and leucine-rich repeat-containing receptors (NLRs), C-type lectin receptors (CLRs), and PYRIN family members (2). Upon DNA virus infection, the cyclic GMP-AMP (cGAMP) synthase (cGAS) senses the viral DNA and catalyzes ATP-GTP transformation to produce the second messenger 2′-3′-cGAMP, which further binds to the stimulator of interferon genes (STING). Subsequently, the STING travels from the endoplasmic reticulum (ER) to the ER-Golgi intermediate compartment (ERGIC) and the Golgi apparatus (3–5). During this process, the carboxy-terminal of STING recruits and activates TBK1, then the activated TBK1 phosphorylates IRF3 (6–9). The phosphorylated IRF3 forms a homodimer, which is then translocated to the nucleus and binds to the interferon stimulus response element (ISRE) of the target genes to induce type I interferon (IFN) transcription (10–12).

Annexins belong to a protein family known for binding and/or holding together with cellular structures on the membranes of various organelles (13). Annexin A2 (ANXA2), also known as annexin II, chromobindin VIII, calpactin I, placental anticoagulant protein IV, lipocortin II, or p36 (13, 14), is one of the important members of the annexin family. Annexin A2 has approximately 98% amino acid sequence similarity among all mammalian species (15). Like other members of the annexin family, Annexin A2 has four annexin repeats, disc-shaped convex structures, which make up a core domain that lies between residues 31 and 338 (16). As a membrane-binding protein, ANXA2 is also a binding partner of F-actin, phospholipids, heparin, and Ca^2+^. Although Annexin A2 was found to differ in its Ca^2+^-binding pattern from other members of the annexin family, its core domain of ANXA2 is generally regarded as a conserved structure (17). Annexin A2 usually exists as a monomer in the cytoplasm or as a complex when it complexes with the members of the S100 protein family (13). For example, the heterotetramer of ANXA2-S100A10 (A2t) consists of two Annexin A2 monomer subunits and two subunits of S100A10, which is usually found on the plasma membrane. Annexin A2 has been proposed to play important roles in exocytosis, endocytosis, cell adhesion, membrane fusion, and membrane trafficking (18). It is well noted that ANXA2 interacts with viral proteins and participates in the viral replication process of a variety of different viruses. For example, ANXA2 interacts with Pseudorabies virus (PRV) US3 and is involved in the PRV virion release process (19). Annexin A2 has been shown to bind directly to cytomegalovirus (CMV) virion and enhance CMV-membrane fusion (20–22). The S100A10 subunit of the ANXA2 heterotetramer facilitates L2-mediated human papillomavirus infection (23). Notably, our recent study demonstrated that ANXA2 also functions as a negative regulator of the RLR signaling pathway during RNA virus infection by disrupting the MDA5-MAVS and MAVS-TRAF3 interactions (24). However, the role of ANXA2 in herpes simplex virus-1 (HSV-1) infection-mediated type I IFN production and its underlying mechanism remain unclear. Given that the cGAS-STING-IRF3 axis is the primary pathway for DNA virus-induced IFN-β production, and that ANXA2 is a membrane-associated protein involved in trafficking, we hypothesized that ANXA2 might regulate the cGAS-STING pathway. Furthermore, as IRF3 is the convergent transcription factor for multiple antiviral pathways, we also sought to investigate whether ANXA2 directly modulates IRF3 activation. In this study, we present biochemical and genetic evidence identifying ANXA2 as a negative regulator of type I IFN production through a dual mechanism: by inhibiting STING Golgi translocation and by disrupting the TBK1/IKKε-IRF3 complex during HSV-1 replication.

## RESULTS

### ANXA2 inhibits type I IFN production independent of S100A10

**Fig 1:**
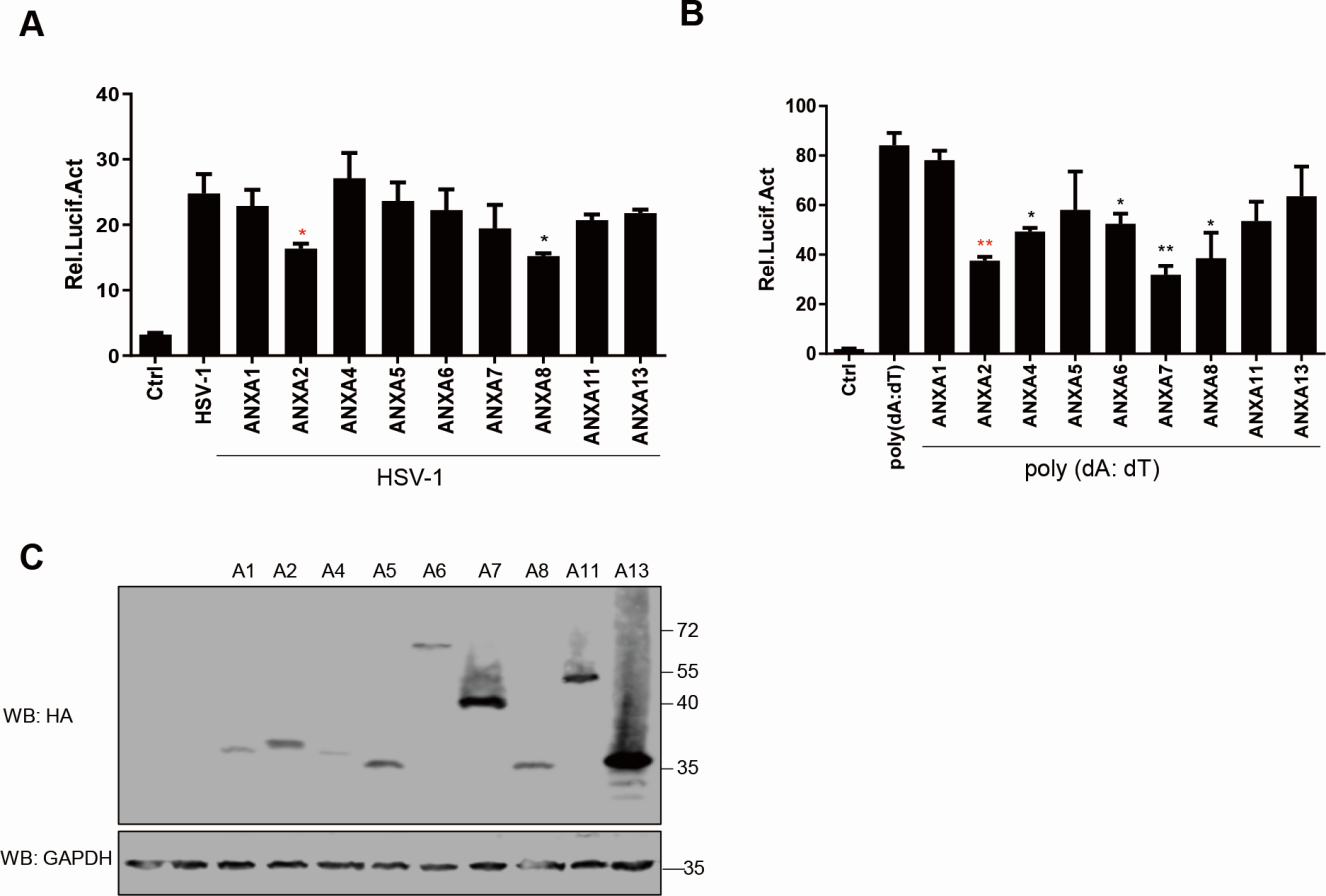
Screening identified ANXA2, a member of the ANXA family, as an inhibitor of type I interferon production. (**A and B**) HeLa cells were transfected with an IFN-β Luc reporter and a Renilla-TK reporter, along with a plasmid encoding one of the ANXA family members, and then infected with HSV-1 (**A**) for 12 h or transfected with poly(dA:dT) (2 μg/mL) for 12 h (**B**). (**C**) The expression levels of ANXA family members and GAPDH were detected by Western blotting at 24 hpi. The Luc activities in the indicated cells were detected. Ns, not significant (*P* > 0.05); *, 0.01 < *P* < 0.05; **, *P* < 0.01; and ***, *P* < 0.001 (one-way ANOVA followed by Bonferroni post-test). Data are representative of three independent experiments with three biological replicates (the mean ± standard deviation [SD] of triplicate assays [panels A to C]) or are representative of three independent experiments with similar results.

The 12 members of the membrane-associated annexin superfamily (ANXA) share a high degree of structural homology and play key roles in mediating a variety of cellular events (25). Some ANXA members are calcium (Ca^2+^)-binding and phospholipid-binding proteins and are involved in viral replication. To identify which ANXA family member regulates innate immunity and thereby inhibits viral replication, we analyzed the impact of nine ANXA family members on HSV-1 infection-induced IFN-β reporter activity using a dual luciferase reporter system analysis. As shown in Fig. 1A through C, ANXA2 and ANXA8 were found to inhibit HSV-1 infection-induced IFN-β reporter activity. Additionally, both ANXA2 and ANXA7 also suppressed poly(dA:dT)-induced IFN-β reporter activity. Since ANXA2 has been implicated in the replication of several DNA viruses (19–22), we selected it to investigate whether it promotes replication by inhibiting host antiviral responses.

**Fig 2:**
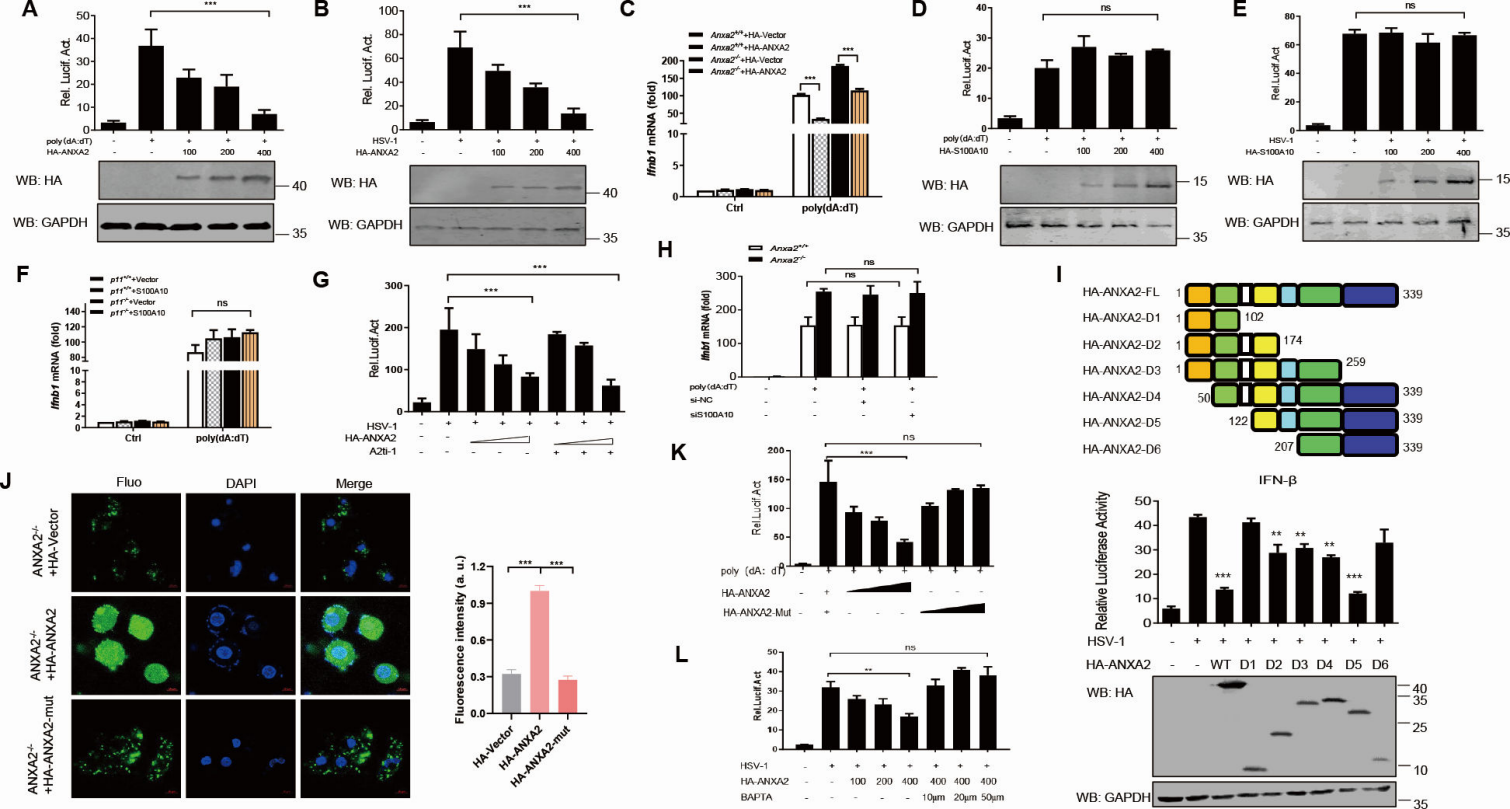
ANXA2 inhibits type I IFN production. (**A and B**) Luc activity of the IFN-β reporter in the HeLa cells transfected with an IFN-β-Luc reporter and a Renilla-TK reporter together with an empty vector or a plasmid expressing ANXA2 and then transfected with poly(dA:dT) (2 μg/mL) (**A**) or infected with HSV-1 (10 multiplicity of infection [MOI]) (**B**) for 12 h. (**C**) Following a 24 h transfection with either the HA-vector or HA-ANXA2 plasmid, HeLa-*Anxa2^+/+^* and HeLa-*Anxa2^⁻/⁻^* cells were stimulated via poly(dA:dT) transfection for 0 or 12 h. Subsequently, the mRNA levels of *IFNB1* were quantified by qPCR analysis. (**D and E**) Luc activity of the IFN-β reporter in the HeLa cells transfected with an IFN-β-Luc reporter and a Renilla-TK reporter together with an empty vector or a plasmid expressing S100A10 for 24 h and then transfected with poly(dA:dT) (2 μg/mL) (**D**) or infected with HSV-1 (10 MOI) (**E**) for 12 h. (**F**) Following a 24 h transfection with either the HA-vector or HA-S100A10 plasmid, CV1-*P11^+/+^* cells, CV1-*P11^⁻/⁻^* cells were stimulated via poly(dA:dT) transfection for 0 or 12 h. Subsequently, the mRNA levels of *Ifnβ1* were quantified by qPCR analysis. (**G**) HeLa cells transfected with an empty vector or a plasmid expressing ANXA2 were infected with HSV-1 for 12 h. Then treated with A2ti-1 (100 μM) for 6 h. (**H**) qPCR analysis of the mRNA levels of *IFNB1* in the HeLa-*Anxa2^+/+^* cells, HeLa-*Anxa2^⁻/⁻^* cells, which were transfected with a shRNA-S100A10, then then transfected with poly(dA:dT) for 12 h. (**I**) ANXA2 and its deleted mutants (top) and Luc activity of the IFN-β-Luc reporter in HeLa cells transfected with an IFN-β-Luc reporter and a Renilla-TK reporter along with a plasmid expressing HA-tagged deleted ANXA2 mutants ANXA2-D1, ANXA2-D2, ANXA2-D3, ANXA2-D4, ANXA2-D5, and ANXA2-D6, and then infected with HSV-1 for 12 h. The expression of plasmids and GAPDH was detected by Western blotting at 24 hpi. (**J**) The calcium ion concentration in HeLa-*Anxa2^⁻/⁻^* cells transfected with HA-Vector, HA-ANXA2-WT, or HA-ANXA2-mut was detected using the Fluo-4 Calcium Assay Kit (Beyotime). Fluorescence intensity was analyzed using Image J software. (**K**) Luc activity of the IFN-β-Luc reporter in HeLa cells transfected with an IFN-β-Luc reporter and a Renilla-TK reporter together with a plasmid expressing ANXA2 or ANXA2 mutant and then stimulated with poly(dA:dT) for 12 h. (**L**) Luc activity of the IFN-β-Luc reporter in HeLa cells transfected with an IFN-β-Luc reporter and a Renilla-TK reporter together with a plasmid expressing ANXA2 and then infected with HSV-1 for 12 h. Cells were treated with a practical inhibitor of ANXA2 and a calcium chelator, respectively. Ns, not significant (*P* > 0.05); *, 0.01 < *P* < 0.05; **, *P* < 0.01; and ***, *P* < 0.001 (one-way ANOVA followed by Bonferroni post-test). Data are representative of three independent experiments with three biological replicates (the mean ± SD of triplicate assays [panels A–L]) or are representative of three independent experiments with similar results.

To investigate the function of ANXA2 in type I production, we first tested whether ANXA2 inhibits IFN-β reporter activity. We found that ANXA2 inhibited poly(dA:dT)-induced and HSV-1-induced IFN-β reporter activity in a dose-dependent manner (Fig. 2A and B). To further confirm these results, HeLa cells with ANXA2 gene deletion (HeLa-*Anxa2^⁻/⁻^*) were generated using CRISPR/Cas9 to further confirm the function of ANXA2. As shown in Fig. 2C, ANXA2 deficiency markedly increased the mRNA levels of *IFNβ1* induced by transfection with poly(dA:dT). Notably, reconstitution of ANXA2 in HeLa-*Anxa2^⁻/⁻^* cells significantly reduced the enhanced *IFNβ1* mRNA levels caused by ANXA2 deficiency (Fig. 2C). ANXA2 usually exists as a heterotetramer when complexes with the S100 protein family (26). We then explored whether S100A10 is involved in IFN-β reporter activity. We found that overexpression of S100A10 did not affect IFN-β production induced by poly(dA:dT) transfection or HSV-1 infection (Fig. 2D and E). Furthermore, S100A10 deficiency does not affect the mRNA levels of *Ifnβ1* induced by transfection with poly(dA:dT) (Fig. 2F). Notably, the mRNA expression level of *Ifnβ1* was also not significantly changed after transfection of *p11^⁻/⁻^* cells with plasmids expressing S100A10 (Fig. 2F). A2ti-1 is a highly selective inhibitor of the A2t complex. It blocks the ANXA2-S100A10 interaction, leading to S100A10 degradation (27). As shown in Fig. 2G, the inhibition of IFN-β by ANXA2 was not affected by the A2ti-1 inhibitor treatment. Consistent with these results, knockout of ANXA2 expression promoted poly(dA:dT)-mediated IFN-β production, while knockdown of S100A10 in HeLa-*Anxa2^⁻/⁻^* using shRNA for S100A10 did not affect IFN-β production compared to HeLa-*Anxa2^⁻/⁻^* cells (Fig. 2H).

ANXA2 comprises three parts: the N-terminal, central structural domain, and C-terminal. The central structural domain of ANXA2 contains type II or type III calcium-binding sites, with ANXA2: 117–161 aa and 201–246 aa being type II binding sites (28). To identify which domain of ANXA2 is required for its inhibition of HSV-1-mediated IFN-β production, the mutants of ANXA2 were co-expressed with IFN-β reporter. We found that ANXA2-D2, ANXA2-D3, ANXA2-D4, and ANXA2-D5 inhibited IFN-β reporter activation during viral infection, but not ANXA2-D1 or ANXA2-D6 (Fig. 2I), suggesting that the core structural domain of ANXA2 (approximately aa 122–174) is involved in inhibiting type I IFN production. To test whether the calcium-binding activity of ANXA2 is related to the inhibition of type I IFN production, we constructed a plasmid expressing an ANXA2 mutant with a mutated calcium-binding site (G50A/G122A/D162A/G207A/E247A/G282A/D322) (28). And we confirmed that this mutant indeed loses its ability to bind Ca²^+^, unlike the wild-type ANXA2 (Fig. 2J). We found that mutating the calcium-binding sites of ANXA2 abrogated its ability to inhibit IFN production (Fig. 2K). Furthermore, treatment with a calcium chelator also suppressed IFN production (Fig. 2L), underscoring the importance of calcium signaling in this process. Together, these results demonstrate that ANXA2 promotes HSV-1 replication by suppressing the type I IFN response.

**Fig 3:**
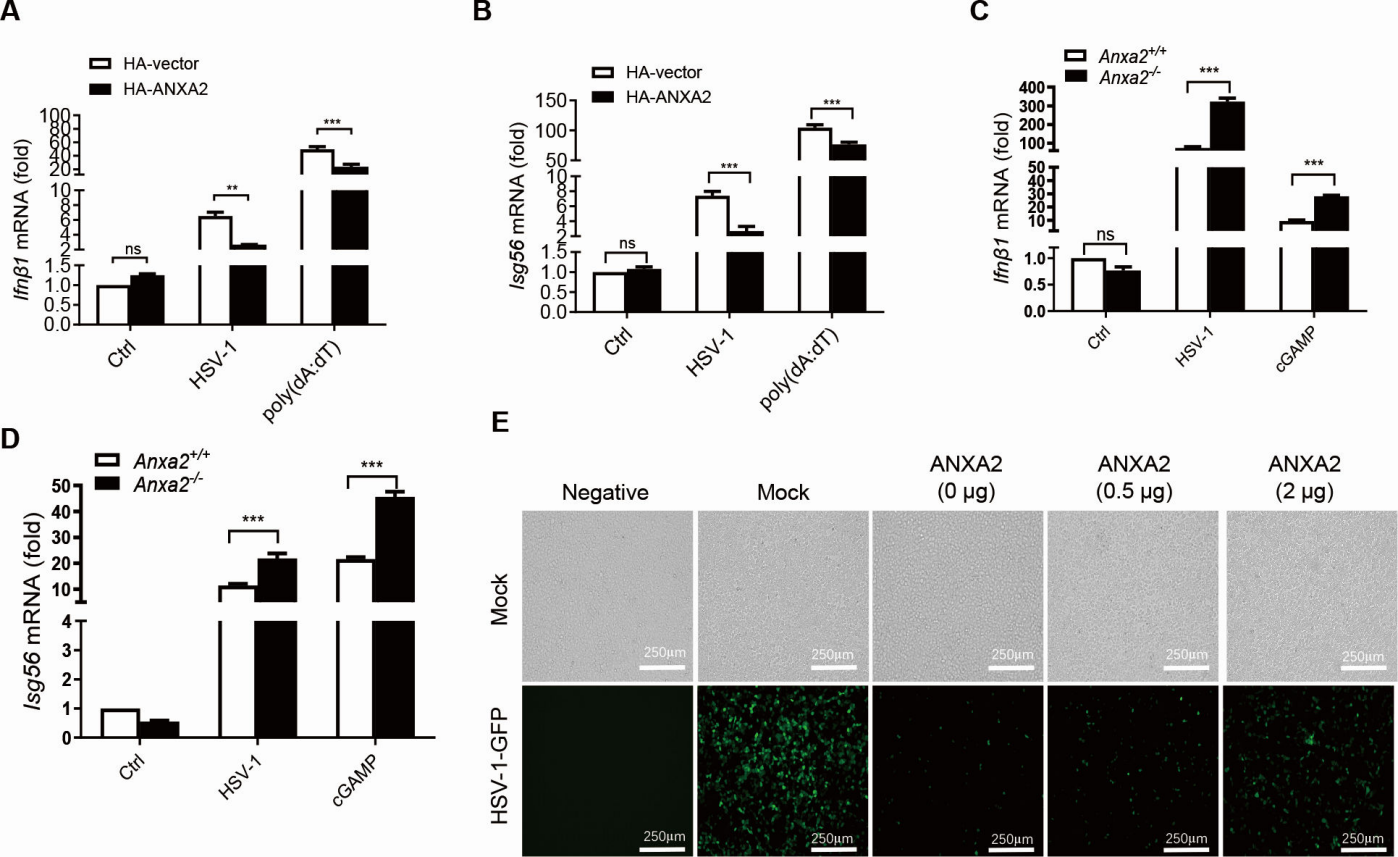
ANXA2 deficiency enhances cellular antiviral responses. (**A and B**) qPCR analysis of *Ifnb1* (**A**) and *Isg56* (**B**) mRNA levels in HeLa cells after transfection with HA-ANXA2 for 24 h followed by HSV-1 infection for 12 h, or after transfection with poly(dA:dT) for 12 h. (**C and D**) qPCR analysis of *IFNB1* (**C**) and *Isg56* (**D**) mRNA levels in HeLa*-Anxa2^+/+^* and HeLa*-Anxa2^⁻/⁻^* cells infected with HSV-1 for 12 h or transfected with cGAMP for 24 h. (**E**) The fluorescence microscope assay was performed to test the inhibitory effects of ANXA2 on GFP-tagged HSV-1 replication. Scale bars, 250 μm. *P < 0.05, **P < 0.01, and ***P < 0.001 (two-tailed Student’s *t*-test). Data are representative of three independent experiments with three biological replicates (mean ± SD [panels A to E]) or are representative of three independent experiments with similar results.

Additionally, we found that overexpression of ANXA2 significantly inhibited the mRNA levels of *IFNβ1* and *Isg56* induced by infection with HSV-1 or transfection with poly(dA:dT) (Fig. 3A and B). In contrast, ANXA2 deficiency significantly increased the mRNA levels of *IFNβ1* and *Isg56* induced by infection with HSV-1 or transfection with cGAMP (Fig. 3C and D). Notably, an IFN sensitivity result showed that the replication levels of HSV-1-GFP were correlated with the expression level of ANXA2 (Fig. 3E). These results suggest that ANXA2 promotes viral replication by inhibiting the production of type I IFN. Taken together, our findings clearly demonstrate that ANXA2, but not A2t, is involved in regulating type I production, which may be related to ANXA2’s function involved in calcium signaling.

### ANXA2 interacted with STING

**Fig 4:**
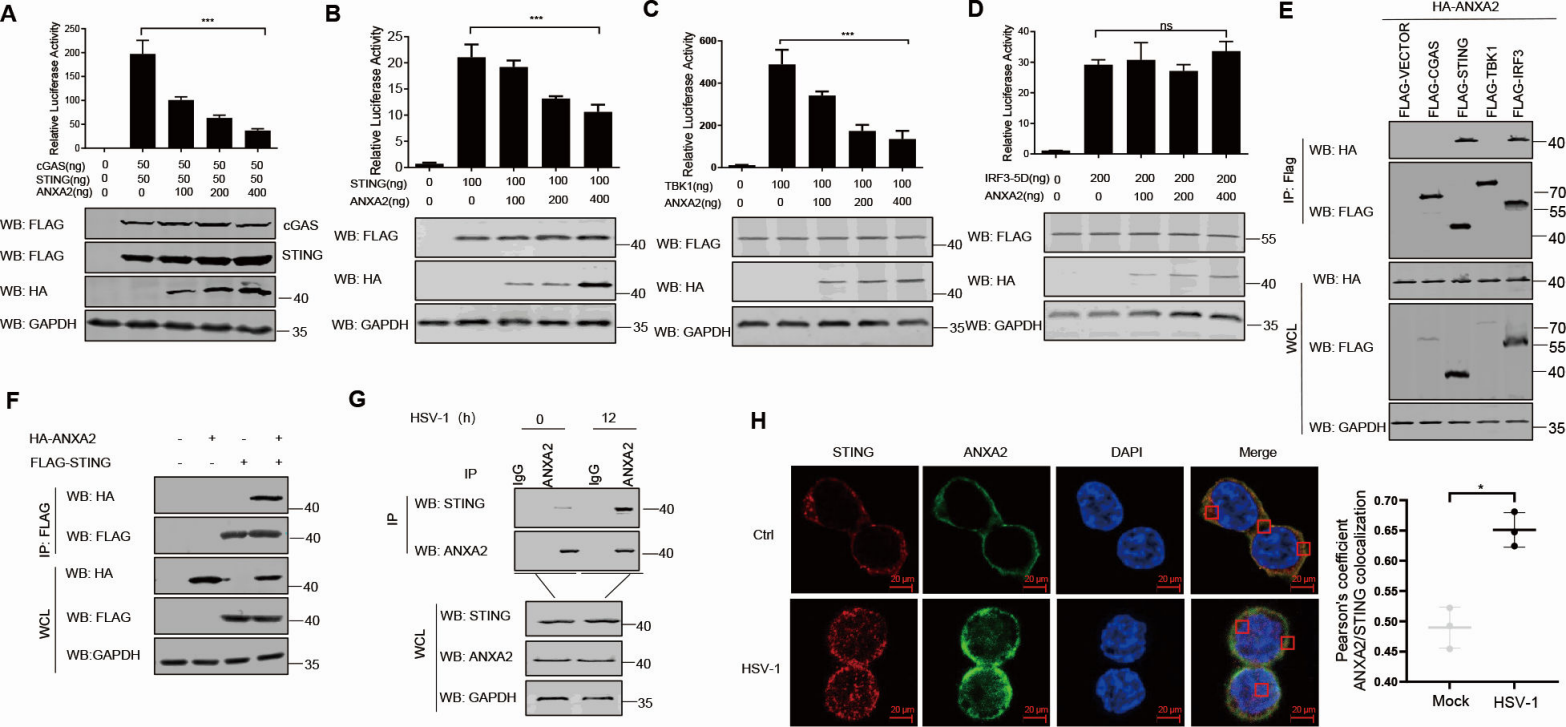
ANXA2 inhibits type I IFN production upstream of IRF3 phosphorylation. (**A–D**) Luciferase activity of the IFN-β-Luc reporter in HeLa cells transfected with an IFN-β-Luc reporter and a Renilla-TK reporter together with a plasmid expressing cGAS + STING (A), STING (B), TBK1 (C), and IRF3-5D (D) along with an empty vector or a plasmid expressing ANXA2. The expressions of cGAS, STING, ANXA2, and GAPDH were detected by Western blotting. (**E**) Co-IP analysis was performed to detect the interaction between ANXA2 and immune molecules in HEK293T cells. HEK293T cells were transfected with a plasmid expressing HA-ANXA2 and a plasmid expressing Flag-tagged cGAS, STING, TBK1, and IRF3. (**F**) Co-IP analysis of the interaction between ANXA2 and STING in HEK293T cells transfected with plasmids expressing HA-ANXA2 and Flag-STING. (**G**) Co-IP analysis of the interaction between endogenous ANXA2 and STING in mouse peritoneal macrophages that were either mock infected or infected with HSV-1 for 0 or 12 h. (**H**) The subcellular localizations of endogenous ANXA2 and STING were analyzed by fluorescence microscopy. HeLa cells were infected with HSV-1 (10 MOI) for 0 or 12 h, and the subcellular localization of ANXA2 and STING in the HeLa cells was detected by confocal microscopy. Scale bars, 20 μm. The Pearson’s correlation coefficient of the images was analyzed using the Zeiss processing system. Ns, not significant (*P* > 0.05); *, 0.01 < *P* < 0.05; **, *P* < 0.01; and ***, *P* < 0.001 (one-way ANOVA followed by Bonferroni post-test). Data represent three independent experiments with three biological replicates (mean ± SD) or three independent experiments with similar results (panels A to F). Panels A and F are derived from data previously presented in our related preprint (bioRxiv, 2021, DOI: https://doi.org/10.1101/2021.12.01.470696).

**Fig 5:**
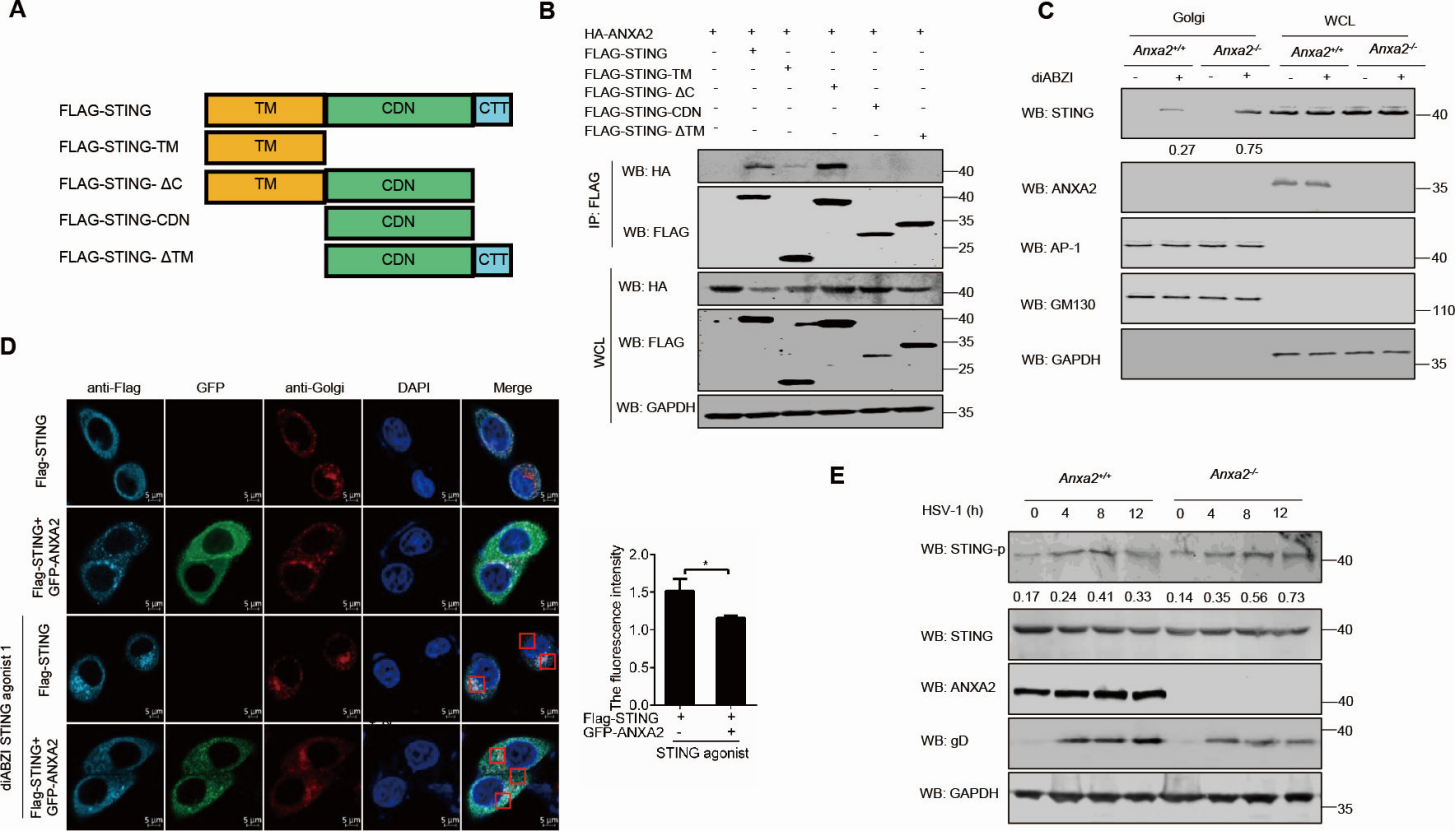
ANXA2 inhibits the localization of STING on the Golgi apparatus. (**A and B**) ANXA2 and its truncation mutants (**A**) and Co-IP analysis of the interaction between ANXA2 and STING or its deleted mutants in HEK293T cells transfected with a plasmid expressing Flag-STING together with vector or HA-ANXA2 and its deleted mutants (**B**). (**C**) The distribution of STING in the Golgi apparatus of HeLa-*Anxa2^+/+^* and HeLa-*Anxa2^⁻/⁻^* cells treated with 1 μM diABZI for 6 h was detected using a Golgi separation assay. The densitometry was performed using ImageJ software, and the band intensities were normalized to their respective loading controls (GAPDH for whole cell lysates, GM130 for Golgi fractions). (**D**) In HeLa cells, Flag-STING was transfected alone or co-transfected with GFP-ANXA2 and Flag-STING, followed by treatment with or without diABZI (1 μM) for 6 h, to examine the subcellular localization of STING by fluorescence microscopy. Scale bars, 5 μm. The quantitative analysis of fluorescence intensity for the indicated signals from the representative images (left). Data are presented as the mean ± SD of fluorescence intensity measured from at least 50 randomly selected cells per condition from three independent experiments. (**E**) Western blotting analysis of STING phosphorylation upon HSV-1 infection in mouse peritoneal macrophages for 0, 4, 8, and 12 h. The densitometry was performed using ImageJ software, and the band intensity of STING-p was normalized to the STING loading control. Data represent three independent experiments with three biological replicates (mean ± SD) or represent three independent experiments with similar results (panels A to E). Panels B and E are derived from data previously presented in our related preprint (bioRxiv, 2021, DOI: https://doi.org/10.1101/2021.12.01.470696).

cGAS-STING plays critical roles in host defense against DNA viruses by inducing type I IFN production via the TBK1/IRF3 pathway (7, 29). To elucidate the underlying molecular mechanisms by which ANXA2 negatively regulates type I IFN production, we first assessed the effect of ANXA2 on the IFN-β reporter activation induced by key molecules in the cGAS-STING signaling pathway. As shown in Fig. 4A through D, ectopically expressed ANXA2 significantly decreased the IFN-β reporter activation induced by cGAS + STING, STING, and TBK1 in a dose-dependent manner, but had no effect on IRF3-5D-induced IFN-β reporter gene activation, suggesting that ANXA2 may inhibit type I IFN production upstream of IRF3 phosphorylation. To identify the target of ANXA2, we examined the interaction between ANXA2 and key molecules in the cGAS-STING signaling pathway. As shown in Fig. 4E, ANXA2 interacted with STING and IRF3 when these proteins were co-expressed with ANXA2 in HEK293T cells. To further confirm the interaction between ANXA2 and STING, HEK293T cells were co-transfected with plasmids expressing Flag-STING and HA-ANXA2. Co-immunoprecipitation (Co-IP) results showed that ANXA2 interacted with STING (Fig. 4F). Additionally, we found that endogenous ANXA2 interacted with endogenous STING in mouse peritoneal macrophages, with or without HSV-1 infection (Fig. 4G). IFA results also revealed that ANXA2 colocalized with STING in the cytoplasm (Fig. 4H). Having established that ANXA2 interacts with STING, we next sought to map the interacting domains. Four different plasmids encoding Flag-tagged STING mutants (STING-TM, STING-ΔC, STING-CDN, and STING-ΔTM) were constructed and transfected with a plasmid expressing HA-tagged ANXA2. The results revealed that ANXA2 interacted with STING-TM and STING-ΔC, but not STING-CDN or STING-ΔTM, indicating that the TM domain of STING is required for the interaction between ANXA2 and STING (Fig. 5A and B).

### ANXA2 inhibits the localization of STING on the Golgi apparatus induced by HSV-1 infection

Previous studies have shown that the translocation of STING from the ER to the Golgi is necessary for STING activation and type I IFN production (30). Therefore, we further explored whether ANXA2 affects the localization of STING in the Golgi apparatus. As shown in Fig. 5C and D, the agonist of STING, diABZI, promotes the transport of STING from the ER to the Golgi (31), while ANXA2 inhibits the diABZI-induced translocation of STING to the Golgi apparatus. It has been reported that the process of translocating STING from ER to the Golgi to activate TBK1, which then phosphorylates STING and the transcription factor IRF3 (32). Therefore, we then examined the effect of ANXA2 on STING phosphorylation and found that ANXA2 deficiency promoted STING phosphorylation (Fig. 5E). Taken together, we demonstrate that ANXA2 inhibits STING translocation to the Golgi, which in turn inhibits STING phosphorylation and ultimately hinders type I IFN production during HSV-1 infection.

### ANXA2 interacts with IRF3

**Fig 6:**
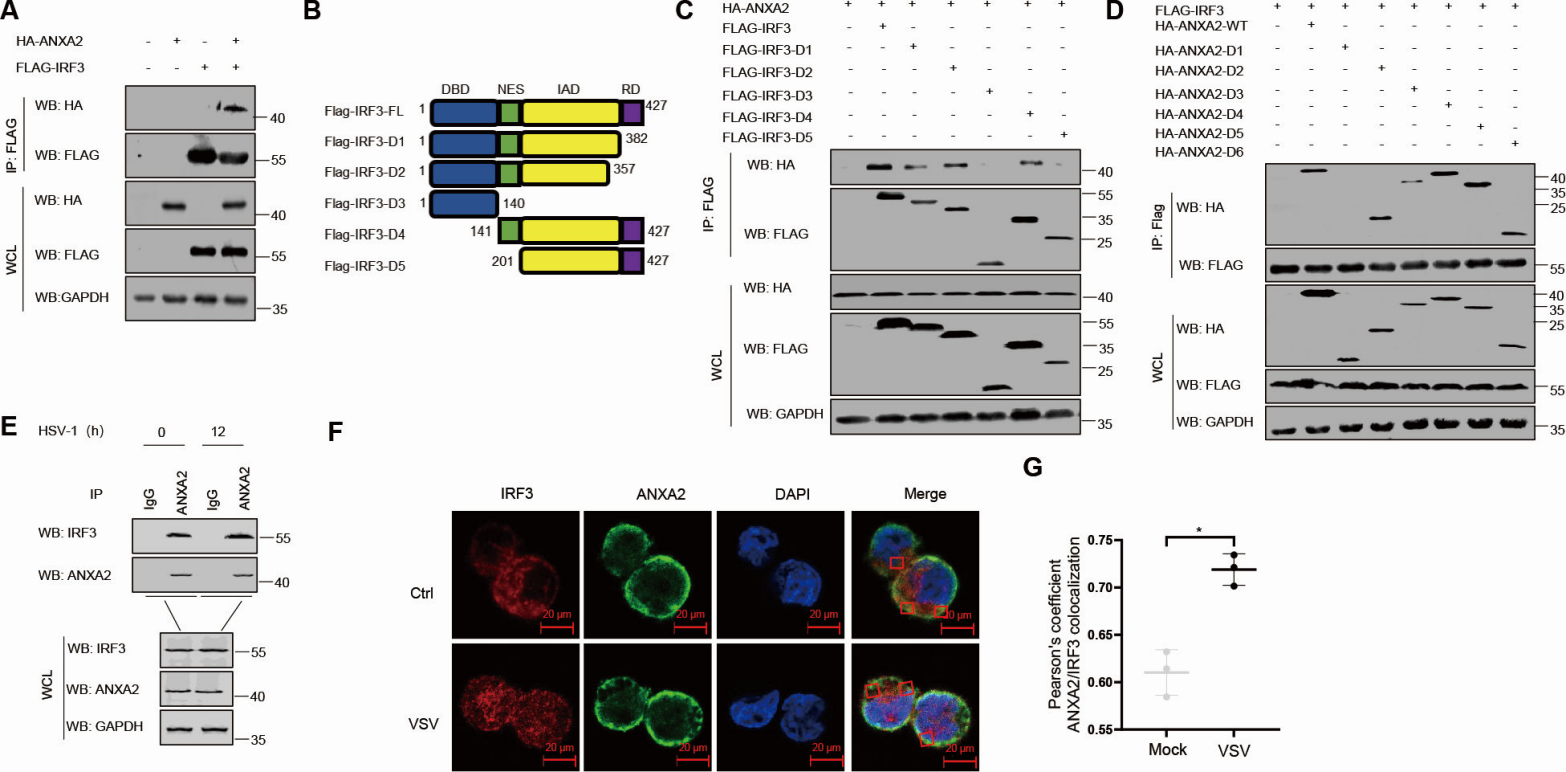
ANXA2 interacts with IRF3. (**A**) Co-IP analysis of the interaction between ANXA2 and IRF3 in HEK293T cells transfected with plasmids expressing HA-ANXA2 and Flag-IRF3. (**B and C**) IRF3 and its deleted mutants (**B**) and (**C**) Co-IP analysis of the interaction between HA-ANXA2 and Flag-IRF3 or its deleted mutants in HEK293T cells. (**D**) Co-IP analysis of the interaction between Flag-IRF3 and HA-ANXA2 or its deleted mutants in HEK293T cells. (**E**) Immunoprecipitation and immunoblot analysis of the interaction of endogenous ANXA2 and IRF3 in mouse peritoneal macrophages infected with HSV-1 at the indicated times. (**F**) The subcellular localizations of endogenous ANXA2 and IRF3 were analyzed by fluorescence microscopy. Scale bars, 20 μm. (**G**) The Pearson’s correlation coefficient of the images was analyzed using the Zeiss processing system. Data are representative of three independent experiments with three biological replicates (mean ± SD in panels A to G). Panels A, C, and D are derived from data previously presented in our related preprint (bioRxiv, 2021, DOI: https://doi.org/10.1101/2021.12.01.470696).

Active TBK1 phosphorylates IRF3 and then triggers type I IFN expression in cells infected with either DNA viruses or RNA viruses (33). In this study, we observed that ANXA2 interacts with IRF3. To confirm the interaction between ANXA2 and IRF3, HEK293T cells were co-transfected with plasmids expressing Flag-IRF3 and HA-ANXA2. Co-IP results showed that ANXA2 interacted with IRF3 (Fig. 6A). IRF3 contains four different domains: an N-terminal DNA-binding domain, an IRF association domain (IAD), a C-terminal regulatory domain, and a nuclear export sequence (NES). To map the regions of IRF3 responsible for its interaction with ANXA2, five different plasmids encoding Flag-tagged deleted IRF3 mutants were constructed, as illustrated in Fig. 6B. Co-IP results revealed that ANXA2 interacted with IRF3-D_1_, IRF3-D_2_, and IRF3-D_4_, but not IRF3-D_3_ or IRF3-D_5_, indicating that the NES domain of IRF3 is required for its interaction with ANXA2 (Fig. 6C). ANXA2 contains two different domains: a C-terminal domain with four repeats and a unique N-terminal domain (13, 26). To map the ANXA2 domain required for the interaction with IRF3, we constructed six different plasmids encoding HA-tagged deleted ANXA2 mutants (Fig. 6D). As shown in Fig. 6E, ANXA2-D_2_, ANXA2-D_3_, ANXA2-D_4_, ANXA2-D_5_, and ANXA2-D_6_ interacted with IRF3, but not ANXA2-D_1_, indicating that the C-terminal region containing the four-repeat domain makes an important contribution to the interaction between ANXA2 and IRF3. Furthermore, endogenous ANXA2 was found to interact with endogenous IRF3 in HEK293T cells infected with HSV-1 (Fig. 6E). Confocal results revealed that ANXA2 colocalized with IRF3 in the cytoplasm (Fig. 6F and G).

### ANXA2 disrupts TBK1-IKKε-IRF3 complex mediated by DNA virus

**Fig 7:**
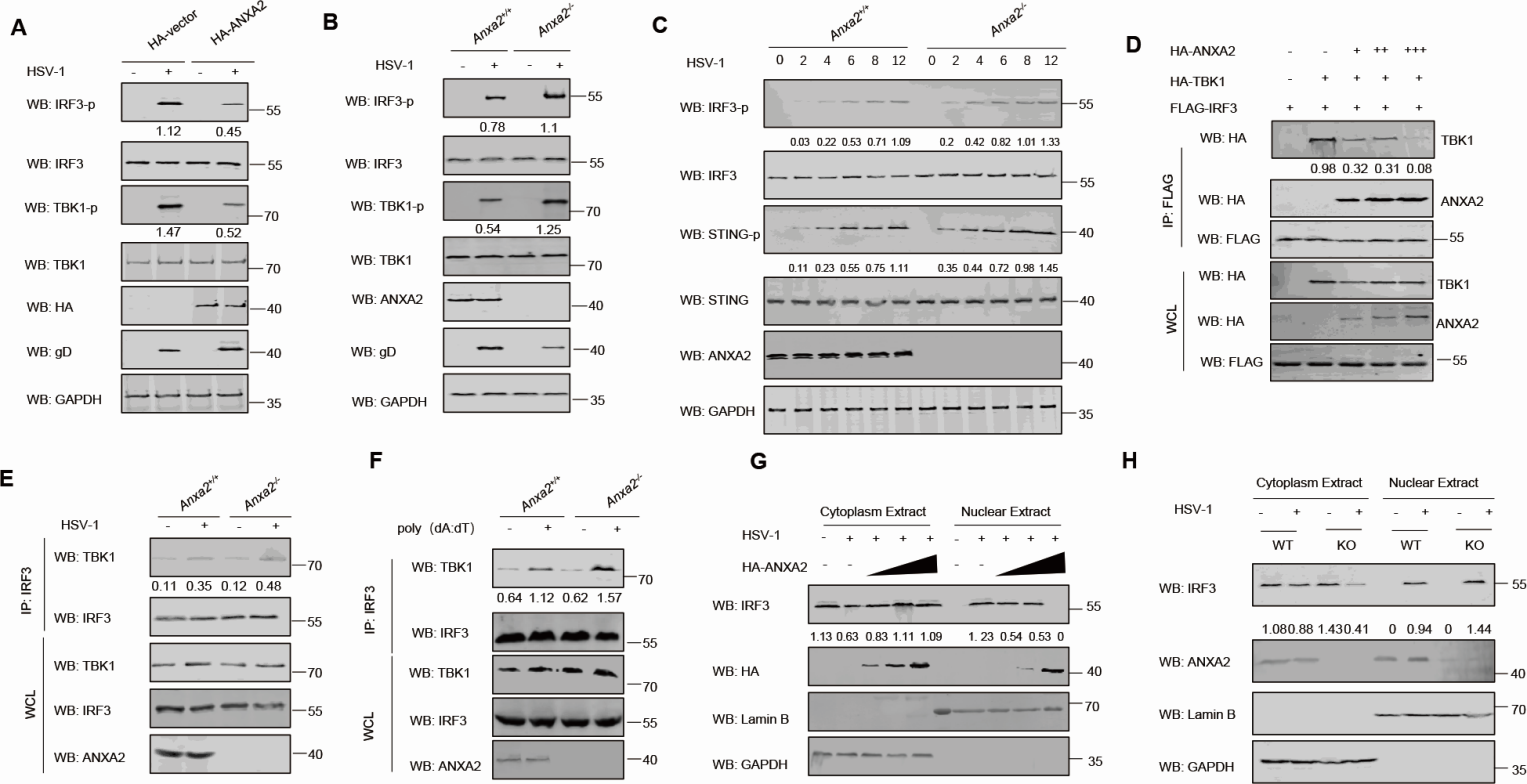
ANXA2 disrupts the TBK1-IKKε-IRF3 complex. (**A**) HeLa cells were first transfected with a plasmid encoding HA-ANXA2 for 24 h and followed by infection with HSV-1 for 12 h. Detection of IRF3, phosphorylated IRF3, TBK1, phosphorylated TBK1, HA-tagged ANXA2, and GAPDH was performed by Western blotting. (**B**) Peritoneal macrophages isolated from *Anxa2^+/+^* and *Anxa2^⁻/⁻^* mice were infected with HSV-1 for 12 h. The expression of IRF3, phosphorylated IRF3, TBK1, phosphorylated TBK1, HA-tagged ANXA2, and GAPDH was then detected by Western blotting. (**C**) Peritoneal macrophages isolated from the *Anxa2^+/+^* and *Anxa2^⁻/⁻^* mice infected with HSV-1 for 0, 2, 4, 6, 8, or 12 h. Detection of IRF3, phosphorylated IRF3, STING, phosphorylated STING, ANXA2, and GAPDH by Western blotting. (**D**) HEK293T cells were transfected with a plasmid encoding Flag-IRF3 alone or together with a plasmid expressing HA-TBK1 and increasing amounts (500 ng, 1,000 ng, and 2,000 ng) of a plasmid encoding HA-ANXA2 as specified. At 24 hpi, Co-IP was performed with anti-FLAG. (**E and F**) Immunoprecipitation and immunoblot analysis of the interaction between endogenous TBK1 and IRF3 in peritoneal macrophages isolated from the *Anxa2^+/+^* and *Anxa2^⁻/⁻^* mice either mock infected or infected with HSV-1 (**E**) or stimulated with poly(dA:dT) (**F**). (**G**) HeLa cells were transfected with increasing amounts (500 ng, 1,000 ng, and 2,000 ng) of a plasmid encoding HA-ANXA2 and then infected with HSV-1. IRF3 in the nuclear and cytoplasmic compartments was detected by Western blotting. (**H**) Peritoneal macrophages isolated from the *Anxa2^+/+^* and *Anxa2^⁻/⁻^* mice were infected with HSV-1. IRF3 in the nuclear and cytoplasmic compartments was detected by Western blotting. Lamin B and GAPDH were used as nuclear and cytosolic markers. For all Western blot analyses, equivalent numbers of cells were plated for each experiment, and equal amounts of total protein (20–30 µg per lane, as determined by BCA assay) were loaded for sodium dodecyl sulfate polyacrylamide gel electrophoresis and immunoblotting. Densitometric analysis was performed using ImageJ software for all results, with band intensities normalized to their respective internal controls (e.g., GAPDH for total cell lysates, Lamin B for nuclear fractions, IRF3-P to IRF3, TBK1-P to TBK1, and STING-P to STING). Data are representative of three independent experiments with three biological replicates (mean ± SD in panels A–G). Panels F and H are derived from data previously presented in our related preprint (bioRxiv, 2021, DOI: https://doi.org/10.1101/2021.12.01.470696).

Previous studies have shown that TBK1 and IKKε are TRAF family member-associated NF-κB activator (TANK)-binding partners, and that the tricomplex (TBK1-IKKε-IRF3) is involved in type I IFN production (34, 35). Active TBK1 and IKKε phosphorylate IRF3, followed by its nuclear translocation and the activation of target gene transcription (36, 37). We subsequently examined the effect of ANXA2 on TBK1 and IRF3 phosphorylation and found that overexpression of ANXA2 in HeLa cells significantly suppressed the phosphorylation levels of TBK1 and IRF3 (Fig. 7A). As shown in Fig. 7B, the phosphorylation levels of TBK1 and IRF3 in macrophages from *Anxa2^⁻/⁻^* mice were higher than those from *Anxa2^+/+^* mice infected with HSV-1, although the total protein levels of IRF3 were not affected. We therefore hypothesized that ANXA2 inhibits IRF3 phosphorylation by disrupting its interaction with TBK1 or IKKε. To further investigate when ANXA2 exerts inhibitory effects on STING and IRF3 during infection, we performed time-course experiments in macrophages from *Anxa2^⁻/⁻^* mice and *Anxa2^+/+^* mice infected with HSV-1. Figure 7C clearly demonstrates that ANXA2 dynamically modulates the phosphorylation of STING and IRF3 as early as 2 h post-infection. As shown in Fig. 7D, overexpressed ANXA2 significantly disrupted the interaction between TBK1 and IRF3 in a dose-dependent manner. Consistent with these results, ANXA2 deficiency significantly increased the interaction of the endogenous TBK1-IRF3 and IKKε-IRF3 upon HSV-1 infection and poly(dA:dT) in the mouse primary peritoneal macrophages from *Anxa2^+/+^* and *Anxa2^⁻/⁻^* mice (Fig. 7E and F). These findings reveal that ANXA2 competes with TBK1 or IKKε in binding to IRF3, leading to the inhibition of IRF3 phosphorylation and its nuclear translocation.

The phosphorylation of IRF3 is related to its translocation to the nucleus. Therefore, we further studied the effect of ANXA2 on IRF3 nuclear translocation. As shown in Fig. 7G, the level of nuclear-translocated IRF3 significantly reduced following ANXA2 expression during HSV-1 infection. Consistent with these results, Western blot results showed that the amount of nuclear translocation of IRF3 induced by HSV-1 infection decreased in a dose-dependent manner with increasing expression of ANXA2, while ANXA2 deficiency significantly enhanced the nuclear translocation of IRF3 upon HSV-1 infection (Fig. 7H). Overall, our findings reveal that ANXA2 inhibits the phosphorylation and nuclear translocation of IRF3 during HSV-1 infection.

### ANXA2 deficiency enhances host antiviral responses *in vivo*

**Fig 8:**
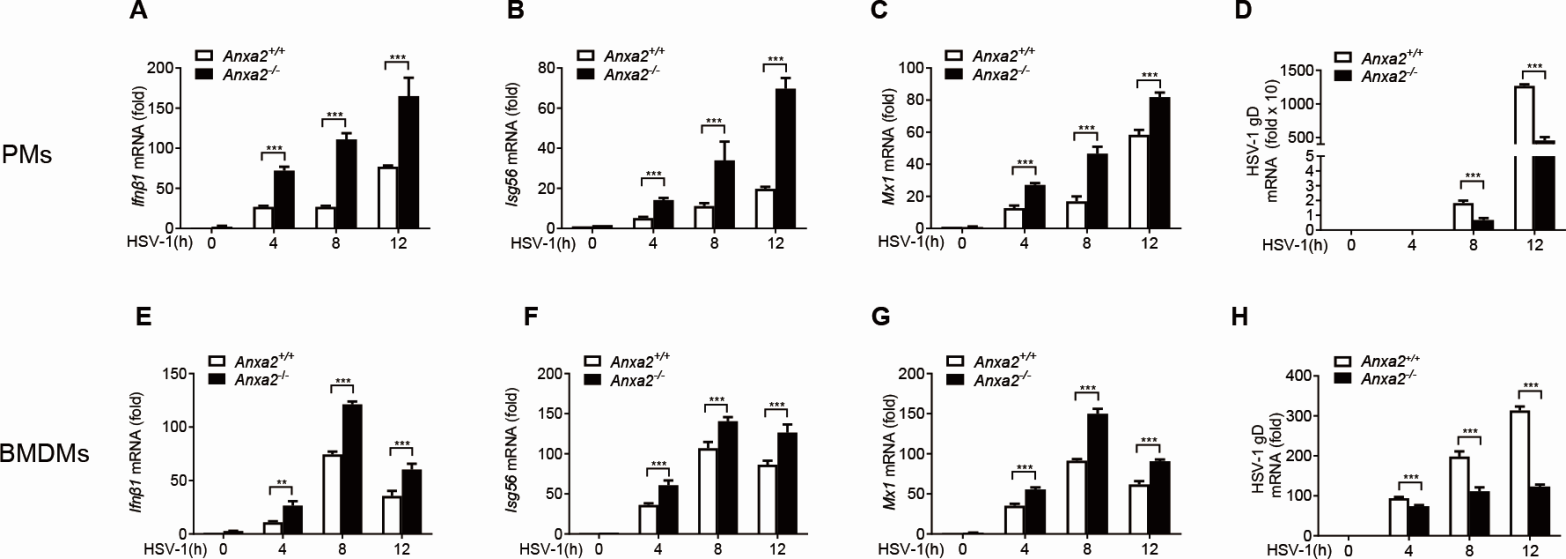
ANXA2 deficiency promotes type I interferon production and thus inhibits viral replication. (**A–C**) qPCR analysis of the mRNA levels of *Ifnβ1* (**A**), *Isg56* (**B**), and *Mx1* (**C**) in *Anxa2^+/+^* and *Anxa2^⁻/⁻^* peritoneal macrophages infected with HSV-1 for 0, 4, 8, or 12 h. (**D**) qPCR analysis of the mRNA levels of the HSV-1 gD gene in *Anxa2^+/+^* and *Anxa2^⁻/⁻^* peritoneal macrophages infected with HSV-1 for 0, 4, 8, or 12 h. (**E–G**) qPCR analysis of the mRNA levels of *Ifnβ1* (**E**), *Isg56* (**F**), and *Mx1* (**G**) in *Anxa2^+/+^* and *Anxa2^⁻/⁻^* BMDMs infected with HSV-1 for 0, 4, 8, or 12 h. (**H**) qPCR analysis of the mRNA levels of the HSV-1 gD gene in the BMDMs isolated from *Anxa2^+/+^* and *Anxa2^⁻/⁻^* mice infected with HSV-1 for 0, 4, 8, or 12 h. *P < 0.05, **P < 0.01, and ***P < 0.001 (two-tailed Student’s *t*-test [panels A to H]). Data are representative of three independent experiments with three biological replicates (mean ± SD in panels A to H).

**Fig 9:**
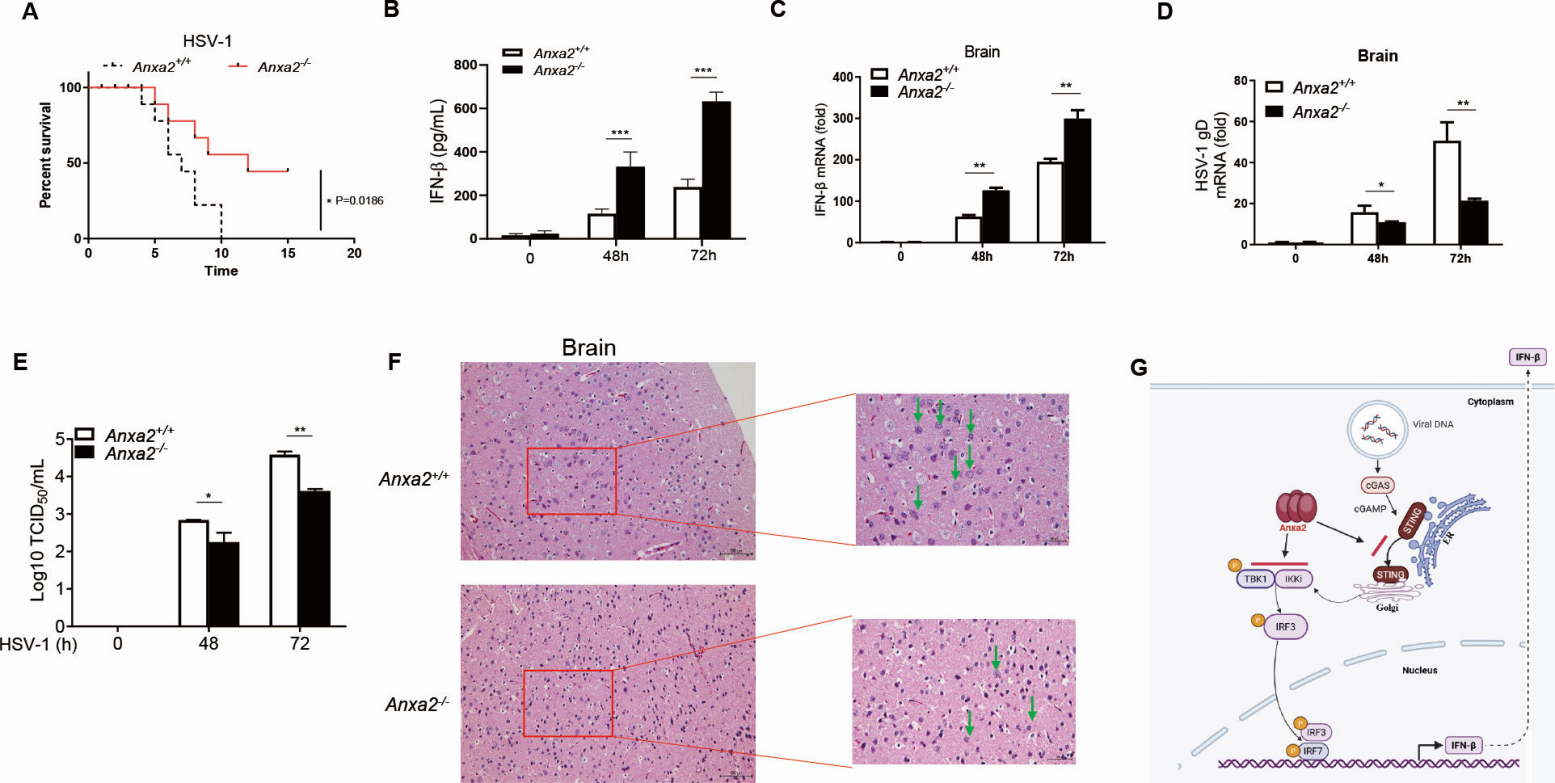
ANXA2 deficiency positively regulates antiviral responses *in vivo*. (**A**) *Anxa2^+/+^* and *Anxa2^⁻/⁻^* mice (10 mice per group) were intraperitoneally injected with HSV-1 (2 × 10^4^ PFU per mouse), and the survival rate of these mice was monitored daily. (**B**) Detection of IFN-β levels in mouse serum from mice by ELISA. (**C**) The mRNA levels of *Ifnb1* in the brain isolated from these mice, as in panel A, were analyzed by qPCR at 48 hpi and 72 hpi; mRNA results are presented relative to those in the uninfected WT mice. (**D**) The mRNA levels of the HSV-1 gD gene in the brain isolated from these mice, as described in panel A, were analyzed by qPCR at 48 hpi and 72 hpi. (**E**) The 50% tissue culture infectious dose (TCID_50_) assay was used to test HSV-1 titer in the brains of mice, as described in panel A. (**F**) Hematoxylin and eosin-stained images of brain sections from *Anxa2^+/+^* and *Anxa2*^⁻/⁻^ mice infected with HSV-1 for 72 h. Scale bars, 50 μm. (**G**) Schematic model of the production of type I IFN inhibited by the ANXA2. *P < 0.05, **P < 0.01, and ***P < 0.001. Data are representative of three independent experiments with three biological replicates (mean ± SD in panels A–F) or are representative of three independent experiments with similar results (in panel F).

To further validate the role of ANXA2 in the production of type I interferons, primary peritoneal macrophages and bone marrow-derived macrophages (BMDMs) were isolated from *ANXA2^⁻/⁻^* mice and their wild-type littermates, respectively. Following HSV-1 infection, *ANXA2^⁻/⁻^* mice macrophages exhibited higher mRNA expression of *Ifnβ1*, *Isg56,* and *Mx1* compared to wild-type mice macrophages (Fig. 8A through C). Furthermore, ANXA2 knockout reduced HSV-1 gD gene mRNA levels post-infection (Fig. 8D). Experiments with BMDMs confirmed the consistency of these findings (Fig. 8E through H). Previous studies have shown that the IFN signal pathway is related to HSV-1 replication. To further define the function of ANXA2 in inhibiting type I IFN production and host antiviral responses *in vivo*, *Anxa2^⁻/⁻^* mice and *Anxa2^+/+^* mice were challenged with HSV-1 through intraperitoneal injections. The results of the mouse survival experiment show that *Anxa2^⁻/⁻^* mice were more resistant to HSV-1 infection than *Anxa2^+/+^* mice (Fig. 9A). Correspondingly, the protein levels of IFN-β in serum from the *Anxa2^⁻/⁻^* mice were also significantly increased (Fig. 9B). Furthermore, we found that the mRNA levels of *Ifnβ1* in the brain of *Anxa2^⁻/⁻^* mice were significantly higher than those of *Anxa2^+/+^* mice after infection with HSV-1 for 48 h and 72 h (Fig. 9C). Consistently, the HSV-1 gD gene mRNA levels in the brains were significantly lower in *Anxa2^⁻/⁻^* mice than those of *Anxa2^+/+^* mice (Fig. 9D). The HSV-1 titers in the brains of the mice*-Anxa2^⁻/⁻^* were significantly lower than their WT littermates after infection with HSV-1 for 72 h (Fig. 9E). Fewer signs of severe inflammation and less pathologic damage were observed in the tissues of *Anxa2^⁻/⁻^* mice compared with WT mice (Fig. 9F). Based on these data, ANXA2 deficiency enhances the host’s antiviral immune response, thereby inhibiting viral replication.

## DISCUSSION

The members of the Annexin family are multifunctional and evolutionarily conserved, which are widely distributed in various tissues and cells of plants and animals. They reversibly bind to phospholipid membranes and calcium ions (Ca^2+^) (38). Previous studies have shown that Annexins play crucial roles in various membrane events regulated by Ca^2+^, such as membrane transport, cytoskeleton formation (39, 40), and ion channel establishment (41–43). Additionally, they are associated with apoptosis and tumorigenesis (44–46), innate immune responses involved in type I IFN production and inflammatory responses (47, 48). Accumulating evidence has shown that annexins are important in regulating host antiviral responses (49). For instance, the C-terminus of ANXA1 directly interacts with TBK1 to enhance the TLRs-mediated IFN-β production in the TLR4/TLR3-TRIF signaling pathway (50). ANXA1 affects the type I IFN production by promoting IFN-β production after RIG-I stimulation. Conversely, knockdown of ANXA1 expression delays the phosphorylation of IRF3 and STAT1, leading to lower expression of ISGs, such as IFIT1 (50). ANXA7 enhances the IFN-β promoter activity induced by chicken MDA5 (chMDA5), thereby inhibiting the infection of the recombinant H5N1 virus (rNS1-SD30) lacking the eIF4GI-binding domain of NS1 (51).

As a membrane scaffold and binding protein, ANXA2 is mainly expressed on the plasma membrane and intracellular vesicles (52, 53), where it participates in the replication process of a variety of viruses. For example, ANXA2 interacts with the non-structural protein 1 (NS1) of AIV to enhance viral replication (54). Previous studies have shown that ANXA2 interacts with the non-structural protein 9 (NSP9) of PRRSV (55) to promote viral replication. Our recent work further established that during RNA virus infection, ANXA2 acts as a negative regulator of the RLR pathway by disrupting the critical interactions between MDA5 and MAVS, and between MAVS and TRAF3 (24). Upon DNA virus infection, the cGAS senses and binds to the viral DNA and catalyzes the formation of 2′-3′-cGAMP, an atypical cyclic di-nucleotide second messenger that can be sensed by STING. STING translocates from ER to the Golgi, leading to phosphorylation of STING. In this study, we noticed that overexpression of ANXA2 inhibited HSV-1-induced IFN-β production, whereas ANXA2 deficiency enhanced type I IFN production and suppressed virus replication *in vitro* and *in vivo*. We found that ANXA2 interacts with the TM domain of STING and inhibits its localization on Golgiosome and phosphorylation. Activated STING recruits and activates TBK1 (56). It is well known that the type I IFN signaling triggered by DNA viruses converges on TBK1. Activated TBK1 phosphorylates IRF3 and promotes its dimerization and translocation into the nucleus, where it forms an active transcriptional complex that binds to the IFN promoter and triggers the type I IFN genes transcription (32, 57).

Normally, IRF3 mainly exists in the cytoplasm and can shuttle between the cytoplasm and the nucleus. The nuclear localization signal (NLS) and nuclear export signal (NES) in IRF3 are constitutively active, with nuclear export being the dominant process. Upon viral infection, active TBK1 and IKKε interact with the IAD of IRF3 and phosphorylate it to facilitate its translocation into the nucleus to induce type I IFN production (11, 58). IRF3 accumulates in the nucleus, and this accumulation depends on the functionality of its NLS (59). Several studies have shown that certain host proteins inhibit IRF3 activation by directly interacting with IRF3. For instance, TRIM26 binds to IRF3 and promotes its K48-linked polyubiquitination and degradation in the nucleus (60). Our previous study also showed that DDX19 inhibits TBK1-mediated and IKKε-mediated phosphorylation of IRF3 by competing with TBK1 or IKKε for binding to the IAD domain of IRF3 (61). In this study, we found that ANXA2 inhibits IRF3 phosphorylation and nuclear entry induced by DNA viruses by impeding TBK1/IKKε/IRF3 complex formation. Unexpectedly, ANXA2 competes with IRF3 to bind to TBK1/IKKε by interacting with the NES domain rather than the IAD domain of IRF3. This phenomenon may be due to steric hindrance related to the structure of ANXA2, which warrants further investigation for confirmation.

The mechanism uncovered here, where ANXA2 disrupts the TBK1/IKKε-IRF3 complex, parallels its role in disrupting the MDA5-MAVS and MAVS-TRAF3 complexes within the RLR pathway (24). This consistent theme—acting as a scaffold to dismantle critical signaling hubs—establishes ANXA2 as a broad-spectrum negative regulator of innate antiviral immunity. The key distinction lies in its pathway-specific targets: in the RLR pathway, it directly binds MDA5 and MAVS, whereas in the cGAS-STING pathway characterized here, its primary targets are STING and IRF3. This dual functionality enables ANXA2 to dampen interferon responses across different viral sensing pathways, potentially creating a cellular environment favorable for diverse viral replication strategies.

ANXA2 is an abundant protein that associates with biological membranes as well as the actin cytoskeleton. It has been implicated in intracellular vesicle fusion, the organization of membrane domains, lipid rafts, and membrane-cytoskeleton contacts. It consists of a highly conserved core domain of four homologous repeats of 70–80 amino acids called the annexin repeats and a unique 30-amino-acid-long N-terminal “head domain” (14, 26). In this study, we found that the amino acid region 122–174 of ANXA2 is required for the inhibition of TBK1-induced IFN-β promoter activation (Fig. 2I). Crucially, we confirmed that a mutant ANXA2 (G50A/G122A/D162A/G207A/E247A/G282A/D322A), which loses its calcium-dependent phospholipid-binding capacity, also abrogates its ability to inhibit IFN production (Fig. 2J and K). This, combined with the effect of a calcium chelator (Fig. 2L), firmly establishes that the inhibition of IFN production by ANXA2 is functionally linked to its calcium ion-binding activity. However, the exact mechanism of action still needs to be further explored.

Previous studies have demonstrated that ANXA2 plays an anti-inflammatory role in response to injury or viral infection (62). Another study demonstrated that ANXA2 plays a role in limiting inflammation by promoting anti-inflammatory signals (63). In line with these findings, mice lacking ANXA2 exhibited a lower survival rate when they were infected with bacteria, indicating a dysregulated inflammatory response (64). Using ANXA2 knockout mice as a model, we demonstrated that IFN-β production significantly increased in primary peritoneal macrophages from *Anxa2^⁻/⁻^* mice upon HSV-1 infection compared to that from wild-type mice, which in turn inhibited viral replication. These data suggest that ANXA2 is a negative regulator of IFN-β production and inflammatory responses during viral infection *in vivo*. Therefore, virus infection may limit IRF3 activation and IFN-β production by inducing ANXA2 expression, thereby promoting its evasion from the host innate immune responses.

In summary, we identified ANXA2 as a novel negative regulator of the interferon response. Mechanistically, ANXA2 negatively regulates IFN-β production by targeting STING and IRF3. ANXA2 disrupts the localization of STING on the Golgi apparatus during DNA virus infection and inhibits TBK1 and IKKε from binding to IRF3 through interaction with IRF3. Together with its established role in the RLR pathway, our findings position ANXA2 as a multifaceted modulator of innate immunity, capable of fine-tuning interferon responses across multiple viral sensing pathways. Understanding these processes may shed light on ANXA2’s new role in viral infection-mediated type I IFN production. Therefore, our study unveils ANXA2 as a potential target for developing immunomodulatory strategies against HSV-1 viral infections.

## MATERIALS AND METHODS

### Mice

*Anxa2^⁻/⁻^* mice generated by homologous recombination technology were purchased from Saiye Biotechnology Co., Ltd. (Guangzhou, China). The mouse genotype was confirmed by PCR using the following primers: forward 5′-CAACTGAGGCACACTCACAAGCG-3′, reverse 5′-GAGAAGGGCTGGCTTAGGGCACT-3′, and 5′-ACTGTGCTGTGAATGCCCACCTTG-3′. All mice were bred in specific-pathogen-free barrier facilities at the Harbin Veterinary Research Institute (HVRI) of the Chinese Academy of Agricultural Sciences (CAAS) in Harbin, China.

### Cell lines

HeLa, HEK293T, and CV1 cells were purchased from the American Type Culture Collection (ATCC, Manassas, VA, USA). HeLa and HEK293T cells were cultured in Dulbecco’s modified Eagle’s medium (DMEM). CV1 cells were cultured in Roswell Park Memorial Institute (RPMI) 1640 medium. All media were supplemented with 10% fetal bovine serum (FBS), 100 U/mL penicillin, and 100 µg/mL streptomycin. Cells were maintained at 37°C in a humidified atmosphere with 5% CO_2_. Primary BMDMs were generated from 6–8-week-old *Anxa2^+/+^*and *Anxa2^−/−^* mice. Mice were euthanized, and femurs and tibias were aseptically dissected. Bone marrow was flushed from the bones using cold RPMI 1640 medium. The collected cell suspension was passed through a 70 µm cell strainer to remove bone fragments and tissue debris. Red blood cells were lysed using ACK lysis buffer (Thermo Fisher Scientific). The remaining progenitor cells were plated in non-tissue culture-treated Petri dishes and cultured in BMDM differentiation medium: RPMI 1640 supplemented with 10% FBS, 20% L929 cell-conditioned medium (as a source of macrophage colony-stimulating factor), 100 U/mL penicillin, and 100 µg/mL streptomycin. Cells were cultured for 7 days, with fresh differentiation medium added on day 3 or 4. On day 7, fully differentiated, adherent BMDMs were detached using cold phosphate-buffered saline (PBS) containing 2 mM EDTA, reseeded into experimental plates, and allowed to adhere for at least 4 h before use in infections or stimulations. Peritoneal macrophages were isolated from mice 3 days after intraperitoneal injection of 3% thioglycollate broth (Merck) and cultured in RPMI 1640 medium supplemented with 10% FBS, 100 U/mL penicillin, and 100 mg/mL streptomycin at 37°C with 5% CO_2_.

### Viruses

HSV-1 strain expressing GFP (HSV-1-GFP) was kindly provided by Prof. Diqiu Liu (HVRI, China). HSV-1 F strain (GU734771.1) (65) was kindly provided by Prof. Chunfu Zheng (University of Calgary, Canada).

### Plasmids

Plasmids expressing Flag-tagged cGAS, STING, TBK1, IRF3, and IRF3-5D have been previously described (66). The IFN-β reporter, ISRE reporter, and TK-Renilla reporter were obtained from Professor Hong Tang (61, 67). To construct plasmids expressing HA-tagged or Flag-tagged ANXA2, cDNAs corresponding to the human ANXA2 gene were amplified by standard reverse transcription-polymerase chain reaction (RT-PCR) using total RNA extracted from HeLa cells as templates and were then cloned into the pCAGGS-HA/Flag vector. All constructs were validated by DNA sequencing. The primers used in this study are available upon request (Table 1).

**TABLE 1:** Primers used for PCR in this study^*a*^

Plasmid	Primers (5'–3')
pCAGGS-ANXA2 (Flag, HA)	F: TGCGAATTCGAGCTCATCGATGGTACCATGTCTACTGTTCACG R: TTAATTAATTAAGATCTGCTAGCTCGAGTCAGTCATCTCCACCA
pCAGGS-HA-ANXA2-D1	F: GAATTCGAGCTCATCGATGGTACCATGTCTACTGTTCACGAA R: TTAATTAAGATCTGCTAGCTCGAGTAGGCCCAAAATCACCGT
pCAGGS-HA-ANXA2-D2	F: TTAATTAAGATCTGCTAGCTCGAGTAGGCCCAAAATCACCGT R: TTAATTAAGATCTGCTAGCTCGAGCAGGGCAACCATCAGCTT
pCAGGS-HA-ANXA2-D3	F: TTAATTAAGATCTGCTAGCTCGAGTAGGCCCAAAATCACCGT R: TTAATTAAGATCTGCTAGCTCGAGCAGGTTCAGGAAAGCATT
pCAGGS-HA-ANXA2-D4	F: GAATTCGAGCTCATCGATGGTACCGGTGTGGATGAGGTCACC R: TTAATTAAGATCTGCTAGCTCGAGTCAGTCATCTCCACCACA
pCAGGS-HA-ANXA2-D5	F: GAATTCGAGCTCATCGATGGTACCGGAACCGACGAGGACTCT R: TTAATTAAGATCTGCTAGCTCGAGTCAGTCATCTCCACCACA
pCAGGS-HA-ANXA2-D6	F: GAATTCGAGCTCATCGATGGTACCGGAACTGATGTTCCCAAG R: TTAATTAAGATCTGCTAGCTCGAGTCAGTCATCTCCACCACA
pCAGGS-Flag-STING-TM	F: GAATTCGAGCTCATCGATGGTACCATGCCCCACTCCAGCCTG R: TTAATTAAGATCTGCTAGCTCGAGGAAATTCCCTTTTTCACA
pCAGGS-Flag-STING-ΔC	F: GAATTCGAGCTCATCGATGGTACCATGCCCCACTCCAGCCTG R: TTAATTAAGATCTGCTAGCTCGAGTTCCTTTTCCTCCTGCCG
pCAGGS-Flag-STING-CDN	F: GAATTCGAGCTCATCGATGGTACCTTCAACGTGGCCCATGGG R: TTAATTAAGATCTGCTAGCTCGAGTTCCTTTTCCTCCTGCCG
pCAGGS-Flag-STING-ΔTM	F: GAATTCGAGCTCATCGATGGTACCTTCAACGTGGCCCATGGG R: TTAATTAAGATCTGCTAGCTCGAGTCAAGAGAAATCCGTGCG

^*a*^ Related to STAR methods. F, forward; R, reverse.

### Viral infection

For qRT-PCR or immunoblot analysis, cells (2 × 10^5^) were plated 24 h before infection with HSV-1 strain F (GU734771.1) at the indicated time points. For viral replication assays, peritoneal macrophages were infected with HSV-1 for 0, 4, 8, and 12 h. Viral replication was analyzed by qRT-PCR. To detect the mRNA level of HSV-gD, total RNA was extracted using TRIzol reagent (Thermo Fisher Scientific, Waltham, MA, USA), and reverse transcription was performed with a PrimeScript RT Reagent Kit (Takara, Tokyo, Japan). Reverse transcription products were amplified using an Agilent-Strata gene Mx Real-Time qPCR system with SYBR Premix Ex Taq II (Takara, Tokyo, Japan) according to the manufacturer’s instructions. Data were normalized to the level of β-actin expression in each individual sample. Relative mRNA expression levels are calculated using the 2^−ΔΔCt^ method. The HSV-gD qPCR primers are listed in Table 2 . For mouse infection, 6–8-week-old age-matched and sex-matched *Anxa2^+/+^* and *Anxa2^⁻/⁻^* littermates were intraperitoneally injected with HSV-1 (2 × 10^7^ PFU per mouse). For survival experiments, the animals’ survival was monitored daily after HSV-1 infection. Sera from HSV-1-infected mice were collected for ELISA analysis at 48 h and 72 h post-infection, and the heart and brain were collected for qRT-PCR, HSV-1 titers, or histological analysis.

**TABLE 2:** Primers used for qPCR in this study

Gene name	Primers (5'–3')
Human IFN-β	F: ATGACCAACAAGTGTCTCCTCC R: GCTCATGGAAAGAGCTGTAGTG
Human β-actin	F: CCTTCCTGGGCATGGAGTCCTG R: GGAGCAATGATCTTGATCTTC
Human ISG56	F: TCATCAGGTCAAGGATAGTC R: CCACACTGTATTTGGTGTCTAG
Human MX1	F: CTCCGACACGAGTTCCACAA R: GGCTCTTCCAGTGCCTTGAT
Mouse IFN-β	F: CCCTATGGAGATGACGGAGA R: CTGTCTGCTGGTGGAGTTCA
Mouse GAPDH	F: AAATGGTGAAGGTCGGTGTGAAC R: CAACAATCTCCACTTTGCCACTG
Mouse ISG56	F: TGCGATCCACAGTGAACAAC R: ACTTCCGGGAAATCGATGAG
Mouse MX1	F: CCTGGAGGAGCAGAGTGACAC R: GGTTAATCGGAGAATTTGGCAA
HSV-1-gD	F: CCATACCGACCACACCGACA R: CGTAGTTGGTCGGTGTAACGCA

### TCID_50_ assay

Vero cells grown in a 96-well plate were infected with 0.1 mL/well of 10-fold serially diluted supernatant or tissue homogenate in quintuplicate. After incubating for 90 min at 37°C, the unattached virus was removed, and DMEM medium supplemented with 2% FBS was added to the Vero cells. Five days post-infection, the TCID_50_ was determined by the Reed-Muench method. All data are shown as the means of three independent experiments.

### Luciferase reporter gene assay

Luciferase activities were measured with the Dual-Luciferase Reporter Assay System (Promega), according to the manufacturer’s instructions. Data were normalized for transfection efficiency by dividing the value of Firefly luciferase activity by the value of Renilla luciferase activity.

### RNA extraction and qPCR

Total RNA was extracted using TRIzol reagent (Invitrogen), and the reverse transcription products were amplified using the Agilent-Strata gene MxReal-Time qPCR system with a PrimeScript RT Reagent Kit (Takara). The reverse transcription products were amplified using an Agilent-Strata gene MxReal-Time qPCR system with TB Green Premix Ex Taq II (Tli RNaseH Plus) (Takara) according to the manufacturer’s instructions. Data were normalized to the level of β-actin expression in each individual sample. The qPCR primers are listed in Table 2.

### Co-immunoprecipitation and Western blot analysis

Co-immunoprecipitation and Western blot analysis were performed as previously described (61). Briefly, for Co-IP, whole cell extracts were lysed in a lysis buffer (50 mM Tris-HCl, pH 7.4, 150 mM NaCl, 5 mM MgCl_2_, 1 mM EDTA, 1% Triton X-100, and 10% glycerol) containing 1 mM PMSF and 1× protease inhibitor cocktail (Roche). Then, cell lysates were incubated with anti-Flag (M2) beads or protein G Plus-Agarose immunoprecipitation reagent (Santa Cruz Biotechnology) together with 1 μg of the corresponding antibodies at 4°C overnight on a roller. The precipitated beads were washed five times with cell lysis buffer. For Western blot analysis, equal amounts of cell lysates and immunoprecipitants were resolved by 10%–12% sodium dodecyl sulfate polyacrylamide gel electrophoresis and then transferred to a polyvinylidene difluoride membrane (Millipore). After incubation with primary and secondary antibodies, the membranes were visualized by ECL chemiluminescence (Thermo Fisher Scientific) or an Odyssey two-color infrared fluorescence imaging system (LI-COR).

### Confocal microscopy analysis

HeLa cells were transfected with the indicated plasmids and then fixed for 20 min in 4% paraformaldehyde in 1× PBS at pH 7.4. The fixed cells were permeabilized for 20 min with 0.3% Triton X-100 in 1× PBS and then blocked in 1× PBS with 10% bovine serum albumin for 30 min. The cells were incubated with the appropriate primary antibodies and then stained with Alexa Fluor 594-labeled goat anti-mouse immunoglobulin G and Alexa Fluor 488-labeled goat anti-rabbit IgG. The subcellular localizations of indicated proteins were observed using a Zeiss LSM-880 laser scanning fluorescence microscope (Carl Zeiss AG, Oberkochen, Germany) with a 63× oil immersion objective. Pearson’s correlation coefficient of the images was analyzed using the Zeiss processing system.

### Image quantitative analysis

Quantitative analysis of fluorescence intensity was performed using ImageJ software (68). Regions of interest (ROI) were drawn around individual cell nuclei or cytoplasm based on the DAPI signal or differential interference contrast image. The mean fluorescence intensity for each channel within the ROI was measured. Background intensity, obtained from an adjacent cell-free area, was subtracted from each measurement. For each experimental condition, at least 50 cells randomly selected from three independent experiments were analyzed. Data are presented as the mean fluorescence intensity ± SD.

### Generation of *Anxa2* knock-out cell lines

HEK293T and HeLa-*Anxa2^⁻/⁻^* cell lines were constructed using the CRISPR/Cas9 method. To create mammalian *Anxa2^⁻/⁻^* cells, one CRISPR guide RNA (sgRNA) sequence targeting the *Anxa2* locus in the genome was chosen based on the specificity scores (http://crispr.mit.edu/). The sgRNA sequence was used as follows: ANXA2 sgRNA, 5′-GCACTGAAGTCAGCCTTATCTGG-3′. Subsequently, the CRISPR/Cas9 system was introduced into HEK293T or HeLa cells using the transfection reagent Lipofectamine 2000. Monoclonal cells were isolated via flow cytometry and validated through sequencing and Western blot analysis. Correctly knocked-out cells were amplified to establish stable knockout cell lines for subsequent experiments.

### IFN sensitivity test

HEK293T cells were transfected with increasing amounts of a plasmid expressing ANXA2 (0 µg, 0.5 µg, and 1 µg) and then infected with SeV for 12 h. The cell supernatants were collected and inactivated with ultraviolet (UV) light. MDBK cells were incubated with the UV-inactivated cell supernatants for 24 h and then infected with HSV-1-GFP (MOI = 1) for 12 h. HSV-1-GFP replication was analyzed under a fluorescence microscope.

### Golgi apparatus isolation

The Golgi apparatus was purified using the Minute Golgi Enrichment Kit (Invent Biotechnologies, Inc., catalog no. GO-037) following the manufacturer’s instructions. The separation efficiency was validated by subsequent Western blot analysis, which demonstrated a significant enrichment of the classical Golgi marker GM130 in the target fraction.

### Statistical analysis

Statistical analysis was conducted using an unpaired Student’s *t*-test, a two-tailed Student’s *t*-test, and one-way or two-way ANOVA followed by the Bonferroni post-test. *P* values <0.05 were considered statistically significant. For mouse survival studies, Kaplan-Meier survival curves were generated and analyzed for statistical significance with GraphPad Prism 6.0. Sample sizes were chosen by standard methods to ensure adequate power, and no exclusions, randomization of weight or sex, or blinding were used for animal studies.

## Data Availability

All relevant data are within the article. The data that support the findings of this study are available from the corresponding author upon reasonable request. Portions of the data presented in this article originated from a preliminary study previously posted by our team (with identical authorship) on the bioRxiv platform (DOI: https://doi.org/10.1101/2021.12.01.470696). The Western blot analyses shown in Fig. 4A and F, Fig. 5B and E, Fig. 6A, C, and D, and Fig. 7F and H are derived from this shared data set. The current article provides a comprehensive analysis, substantial new experimental validation, and expanded conclusions not included in the preliminary report.
